# Health Literacy and Primary Prevention of Cardiovascular Disease: A Scoping Review

**DOI:** 10.1177/00333549251322649

**Published:** 2025-06-09

**Authors:** Bonnie Beasant, Kara Anderson, Georgie Lee, Mojtaba Lotfaliany, Monica Tembo, Scott McCoombe, Vanessa Vaughan, Julie A. Pasco, Sarah M. Hosking

**Affiliations:** 1 School of Medicine, Institute for Mental and Physical Health and Clinical Translation (IMPACT)–Barwon Health, Deakin University, Geelong, Australia; 2 Medical School, The University of Western Australia, Perth, Australia; 3 WA Centre for Health and Ageing, University of Western Australia, Crawley, Australia; 4 School of Medicine, Deakin University, Geelong, Australia; 5 Department of Medicine–Western Health, The University of Melbourne, St Albans, Australia; 6 Department of Preventive Medicine, Monash University, Melbourne, Australia

**Keywords:** health literacy, cardiovascular disease prevention, scoping review

## Abstract

**Objectives:**

Although cardiovascular disease (CVD) is responsible for a large global burden of disease, a large proportion of CVD incidence can be prevented through health literacy (ie, the skills and resources of an individual to access, understand, and use information to make decisions and act on one’s own health and health care). We reviewed and synthesized peer-reviewed literature on health literacy and primary prevention of CVD.

**Methods:**

We followed methods from the review’s previously published protocol, which outlined a search strategy conducted on August 16, 2024, for 6 databases, linking concepts of health literacy and CVD risk and its associated knowledge, attitudes, or practices. One reviewer screened and extracted all articles, and a second reviewer screened a randomly selected 10% of articles at each stage to examine interrater agreement. We used the Office of Health Assessment and Translation Risk of Bias Tool to assess the potential risk of bias.

**Results:**

Of 35 studies in the synthesis, 26 (74%) were cross-sectional and 21 (60%) measured functional health literacy only. Twenty-three articles investigated health literacy as an exposure variable, 20 of which reported significant results. Eight articles examined the administration of health literacy interventions to populations at risk of CVD, and 4 presented health literacy profiles of populations at risk of CVD. Each study demonstrated at least 1 area of potential risk of bias but was deemed low risk of bias overall.

**Conclusions:**

Several studies in this review found an association between health literacy and CVD risk. More longitudinal studies, as well as studies that measure health literacy more deeply than simply reading and comprehending health texts, are needed to better understand the extent of this relationship.

Cardiovascular disease (CVD) was responsible for 17.9 million deaths worldwide in 2019.^1^ Although CVD represents a range of conditions with varying causality, 85% of CVD deaths in 2019 were due to ischemic heart disease or stroke, of which risk can be partially attributed to modifiable lifestyle factors (eg, alcohol and tobacco use, physical inactivity, poor diet).^1^ Other risk factors include elevated blood pressure, blood glucose, lipids, and body weight, which may be managed through pharmacologic methods, as well as unmodifiable risk factors such as sex and age. CVD prevention involves targeting modifiable elements to reduce risk.

Several tools have been developed to calculate an individual’s likelihood of a CVD event (eg, ischemic heart disease, stroke) in the next 10 years, often presented as a composite score with varying cutoff points for low or high risk of a cardiovascular event.^2,3^ In addition to research on CVD risk, a large body of research has investigated awareness, beliefs, and attitudes toward CVD in healthy populations, often presented in categories such as perceived risk, perceived susceptibility, or results of objective knowledge tests.^4-6^ Perceptions and attitudes toward CVD are needed for a comprehensive understanding of behavioral risk despite less objectivity than risk of CVD event.^7,8^ Although CVD risk can be modified through individual behaviors, several upstream factors may act as barriers to an individual’s ability to optimize these behaviors, including socioeconomic status, living and working environments, and cultural factors.^1^ Indeed, a social gradient of CVD deaths was observed as early as 1984 by Marmot et al in a study of 17 530 civil servants, revealing that social class was inversely related to CVD mortality, even after adjusting for factors commonly used in CVD risk calculations.^9^

Health literacy has been identified as a potential mediator between socioeconomic disparities and lifestyle behaviors, which may position it as a facilitator of preventive action for CVD.^10^ Health literacy is defined as “the skills and resources of a person to access, understand, and use information to make decisions, and take action on their own health and healthcare.”^11,12^ As a field, health literacy hosts various conceptualizations, definitions, and measurement tools.^11,13-17^ As well as reflecting individual ability, health literacy encompasses interpersonal and systemic supports of an individual, community, or population. Individual health literacy can be conceptualized into 3 levels: *functional*, representing basic literacy and comprehension skills; *communicative*, representing interpersonal skills and resources; and *critical*, representing the ability to critically analyze and apply information to health decisions.^18^

Decreased health literacy is associated with a range of suboptimal health outcomes, including increased hospitalizations and emergency department visits,^19^ decreased self-management of chronic diseases,^20,21^ and decreased medication adherence.^19,22^ These outcomes extend to populations already diagnosed with CVD^23,24^; however, to our knowledge, no synthesis to date has assessed the association between health literacy and objective measures of CVD risk. Similarly, to our knowledge, research has not previously been summarized to investigate the association between health literacy and attitudes, beliefs, and knowledge of CVD prevention. While some research investigated health literacy and CVD prevention practices, this research occurred as discrete silos of prevention elements. For example, a review by Isa et al reported a positive association between health literacy and blood pressure control.^25^ Yet, a comprehensive review of all CVD prevention literature in the health literacy field, including health literacy’s association with CVD risk scores, may illuminate areas for further investigation and synthesize data into a widely accessible evidence base. Thus, the aim of this review was to synthesize peer-reviewed literature concerned with health literacy and primary prevention of CVD.

## Methods

The research team deemed a scoping review the most appropriate strategy to address this research question because of an anticipated paucity of literature on health literacy and CVD, as well as the heterogenous use of health literacy assessment tools across the field, which made a meta-analysis infeasible.^26^ Furthermore, initial searches suggested that the literature was also heterogenous in study design and research aims. We conducted a sensitive search strategy, opting for more studies of broader relevance as opposed to fewer studies of specific relevance. Our search consisted of 6 databases (Medline, Global Health, PubMed, Embase, PsycINFO, and CINAHL), linking relevant terms for health literacy, CVD, knowledge, behavior, and prevention. Full search terms are available in the published protocol.^27^ We searched databases and imported results to Covidence software on August 16, 2024. Studies met inclusion criteria if they were published in English, were concerned with primary prevention of CVD, and included a measure of health literacy in their analysis. We tabulated non-English full texts with abstracts that were otherwise eligible. We excluded theses, opinion pieces, conference proceedings, and studies with nonlayperson populations (ie, health professionals). We also excluded studies published before 2014.

We conducted a pilot screening of 25 randomly selected articles, from which 2 raters at each stage (B.B., G.L. at abstract; B.B., K.A. at full text) reached 100% agreement and refined inclusion and exclusion criteria. The second reviewers from each stage (G.L. and K.A.) then assessed 10% of imported abstracts and full texts, as extracted via a modified Covidence template, as well as the risk of bias from 10% of the full texts to ensure adherence to protocol, balancing the need for dual review with insufficient resources for complete dual review. We used the Office of Health Assessment and Translation (OHAT) Risk of Bias Tool on all included studies, where elements of each article are judged on a scale of definitely low risk of bias to definitely high risk of bias.^28^ A numeric value for each OHAT criterion was assigned, where 0 = definitely low risk of bias, 1 = probably low risk of bias, 2 = probably high risk of bias, and 3 = definitely high risk of bias. With 7 criteria for bias (and a further 5 criteria for experimental designs), overall risk-of-bias scores may range from 0 to 21. We conducted a narrative review to identify thematic categories of studies. We provide a PRISMA-ScR overview (Preferred Reporting Items for Systematic Review and Meta-analyses for scoping reviews) in the Figure.^29^ Deakin University’s ethics committee does not require an ethics review or approval for a literature review article.

**Figure.**
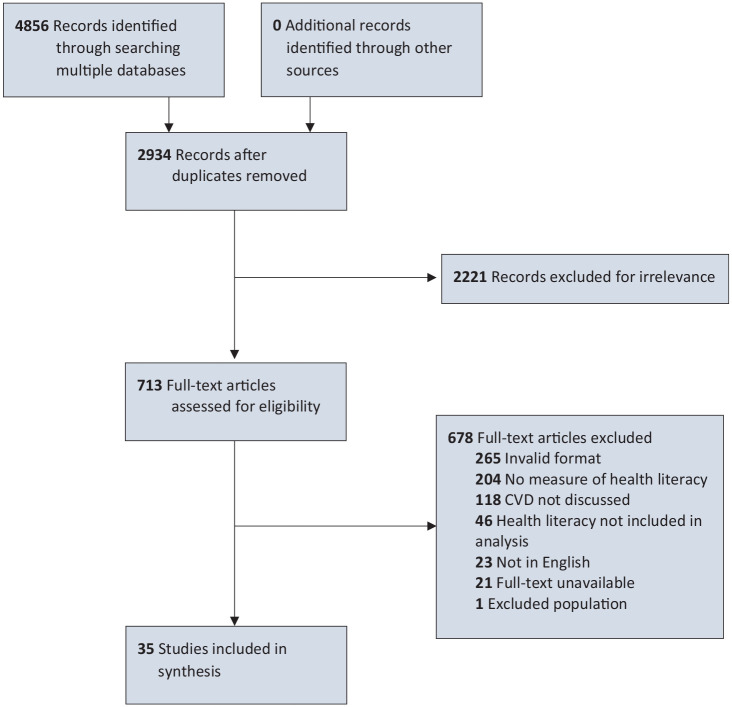
PRISMA-ScR diagram of studies on health literacy and primary prevention of cardiovascular disease. Abbreviations: CVD, cardiovascular disease; PRISMA-ScR, Preferred Reporting Items for Systematic Reviews and Meta-analyses for scoping reviews.

## Results

We excluded 23 studies because the full text was not available in English. Of these 23 studies, the most common languages were Chinese (n = 6), Korean (n = 5), Persian (n = 4), and Spanish (n = 4). After interrater agreement reached >80% at all stages, we included 35 articles for extraction; a manual search of their reference lists yielded no further results.^30-64^ Three major themes of study designs emerged: those that investigated the role of health literacy in CVD risk (n = 23), intervention programs (n = 8), and studies describing populations at risk of CVD (n = 4).

Most studies were cross-sectional (74%) and typically recruited participants from a health service (60%) (Table 1). Most studies took place in North America (31%) and Asia (31%). The number of participants ranged from 23 to 3719. All 35 studies measured functional health literacy, 11 measured critical health literacy, and 3 measured communicative health literacy. Most studies had more female than male participants (71%); mean participant ages ranged from 33 to 75 years. Of the 25 studies that provided a definition of health literacy, 10 used a US definition from 2004: “the degree to which individuals have the capacity to obtain, process, and understand basic health information and services needed to make appropriate health decisions”^67^ (Table 2). This definition and that by Schillinger et al were the only ones to include health services.^73^ Other definitions used by studies were limited to health information or health issues.^11^

**Table 1.** Characteristics of studies on health literacy and primary prevention of cardiovascular disease, 2024 (N = 35)

Characteristic	No. (%)
Study design	
Cross-sectional	26 (74)
Nonrandomized experimental	3 (9)
Randomized controlled trial	4 (11)
Pilot	1 (3)
Mixed methods	1 (3)
Recruitment setting	
Patient populations	23 (66)
Convenience samples	7 (20)
Randomly sampled	4 (11)
Existing study	1 (3)
Location	
United States	11 (31)
Iran	5 (14)
Australia	4 (11)
China	3 (9)
Asia, other than China	6 (17)
Europe	6 (17)
Publication year	
2014-2016	8 (23)
2017-2019	9 (26)
2020-2022	11 (31)
2023-2024	7 (20)
Health literacy category measured	
Functional	35 (100)
Communicative	3 (9)
Critical	14 (40)
Risk factors measured	
Blood pressure	14 (40)
Tobacco use/smoking status	13 (37)
Attitudes toward cardiovascular disease	13 (37)
Body mass index	12 (34)
Self-reported physical activity	9 (26)
Cholesterol	8 (23)
Diabetes status	8 (23)
Self-reported diet	8 (23)
Medication adherence	7 (20)
Participant age, y, mean (SD)	
Youngest	32.2 (13.3)
Oldest	78.6 (12.1)
Sample size	
<100	4 (11)
100 to 199	8 (23)
200 to 499	12 (34)
500 to 999	4 (11)
1000 to 1999	4 (11)
2000 to 3500	3 (9)

**Table 2.** Studies on health literacy and primary prevention of CVD, 2024

Author (year)	Design, population (country)	No. of participants	Health literacy tool, elements measured	Definition of health literacy	Other measures	Key findings
Adam (2023)^30^	Cross-sectional, cohort participants (United States)	3719	Author adapted, functional	Personal health literacy is the degree to which individuals have the ability to find, understand, and use information and services to inform health-related decisions and actions for themselves and others.^65^	Sociodemographic, CVD risk (American Heart Association’s Life Simple)^66^	Health literacy was inversely associated with CVD risk and blood glucose control after adjustment.
Aranha (2015)^31^	Cross-sectional, older patients seeking care at a health care facility (United States)	150	Shortened TOFHLA, functional	The degree to which individuals have the capacity to obtain, process, and understand basic health information and services needed to make appropriate health decisions.^67,68^	Sociodemographic, blood pressure, glucose, HbA_1c_ and lipids, presence of hypertension and diabetes, nutritional literacy, BMI, tobacco use, and CVD risk factors (American College of Cardiology practice guidelines)^69^	Health literacy did not predict CVD risk factors.
Araújo (2018)^32^	Cross-sectional, patients who were hypertensive or diabetic from a cluster of health care centers (Portugal)	401	HLS-EU, functional, critical	The ability to access information on health issues and understand, interpret, and evaluate it to make decisions on these issues.^11,70^	Sociodemographic, health conditions, health service use	Health literacy difficulties included judging illness information in the media; weighing treatment options; judging how to prevent and manage CVD risk factors; finding screening information; understanding food packaging; and understanding the effects of the workplace, neighborhood, and politics on health
Bonner (2022)^33^	Randomized controlled trial, adults (Australia)	859	NVS, functional	None provided	Sociodemographic, CVD risk (Framingham Risk Score),^71^ heart age, knowledge of own cholesterol and blood pressure levels, diet, exercise, and smoking	No differences in intentions immediately after intervention, risk perception, emotional response, or decisional conflict. No significant differences after 4 wk other than the literacy-sensitive group ate more fruit and the intervention group was more likely to know its risk than the control group. Within the intervention group, health literacy was associated with self-reported calls to the Heart Foundation helpline and verbatim knowledge of CVD percentage risk at follow-up.
Cheng (2018)^34^	Cross-sectional, patients of a health care center (Taiwan)	1010	Short-form Mandarin Health Literacy Scale, functional	The ability to communicate and understand basic health information to make appropriate health decisions concerning health care and disease prevention.^72^	CVD risk (Framingham Risk Score)^71^	Inverse association between health literacy and CVD risk, BMI, and fatty liver disease, as well as metabolic syndrome in women
Crook (2016)^35^	Cross-sectional, patients of a regional health center in Texas (United States)	180	NVS, functional	Functional health literacy is a measure of a patient’s ability to perform basic reading and numerical tasks required to function in the health care environment and is distinct from education level and language ability.^73^	Internet use, perceived knowledge of heart health, information overload, information sharing, attitude toward information, behavioral intentions	Health literacy was positively associated with attitude toward information sharing and behavioral intentions. Higher internet use predicted health literacy. Perceived heart health knowledge was correlated with health literacy. Higher health literacy predicted poorer attitudes toward healthy heart information in study. Health literacy predicted 11% of variance in overall model.
Darvishpour (2022)^36^	Cross-sectional, older adults with hypertension admitted to hospital (Iran)	150	HELIA, functional, critical	None provided	Sociodemographic, self-efficacy, hypertension self-care, duration of hypertension, sources of health information	Health literacy predicted self-efficacy and self-care behaviors and contributed to 40% of variance in self-care behaviors but only 6.2% of variance in self-efficacy.
Debussche (2018)^37^	Cross-sectional, patients of education services for people with high CVD risk (France)	175	Health Literacy Questionnaire, functional, communicative, critical	The ability to communicate and understand basic health information to make appropriate health decisions concerning health care and disease prevention.^72^	Sociodemographic, health conditions	Strengths were feeling supported by health care provider and understanding health information enough to know what to do. Challenging areas were having sufficient information to manage health, having social support for health, and navigating the health care system.
Enjezab (2021)^38^	Cross-sectional, women aged 40-60 y (Iran)	280	HELIA, functional, critical	The degree to which individuals have the capacity to obtain, process, and understand basic health information and services needed to make appropriate health decisions.^67,68^	Sociodemographic, weight, way of accessing health information, perceived susceptibility, and perceived severity of CVD	Mean perceived risk of CVDs was moderate. Positive association between accessibility domain of health literacy and perceived risk of heart disease.
Fajardo (2024)^39^	Randomized controlled trial, adults aged 45-74 y (Australia)	546	Single Item Literacy Screener, functional	The skills that enable individuals to obtain, understand, and use information to make decisions and to take actions that will have an impact on health status.^74^	Sociodemographic, intention to attend heart health check, psychological response to text prompt	Significant interaction effects were observed for intention to receive a heart health check after text by health literacy level.
Faruqi (2015)^40^	Nonrandomized experimental study, patients and general practitioners/practice nurses of 4 Inner West of Sydney clinics (Australia)	113	Adapted Single Item Literacy Screener, functional	The capacity to acquire, understand, and use information for health.^75^	Clinic-level audits, baseline and follow-up of patients who did not have a chronic disease: blood pressure, lipids, glucose, smoking, alcohol, waist, BMI, antihypertensive, lipid-lowering medication, 5-y absolute CVD risk	Nonsignificant trends for improvement in clinic recording of risk factors. Little change in the proportion of patients at risk between baseline and 4 mo. Nonsignificant trend toward improvement of general practitioner health literacy practices. After intervention, providers recognized the need for a special effort to communicate with patients with low health literacy levels and had a greater appreciation of the importance of printed materials.
Fu (2022)^41^	Randomized controlled trial, patients with uncontrolled hypertension (Hong Kong)	289	Chinese Health Literacy Scale for Chronic Care, functional	None provided	Sociodemographic, home blood pressure monitoring knowledge and frequency of use, smoking, blood pressure, BMI	Low health literacy group had mean weight loss in group intervention; control group with lower health literacy levels had mean weight gain. Adequate health literacy was a predictor for higher home monitoring knowledge.
Gaffari-Fam (2020)^42^	Cross-sectional, adults aged ≥30 y with hypertension, living in suburban northwest Iran (Iran)	210	Select HELIA scales, functional, critical	None provided	Sociodemographic, lifestyle (psychological, weight control, nutrition, physical activity, drug avoidance)	Access and decision-making domains of health literacy were associated with healthy lifestyle. Health literacy explained 33.9% of variance in lifestyle behaviors. Lifestyle and health literacy explained 21.7% of the variance in hypertension.
Halladay (2017)^43^	Norandomized experimental study, patients with uncontrolled hypertension from 6 primary care practices (United States)	493	Shortened TOFHLA, functional	The degree to which individuals have the capacity to obtain, process, and understand basic health information and services needed to make appropriate health decisions.^67,68^	Sociodemographic, blood pressure, BMI, use of medications, disease status, smoking, comorbidities, medication adherence, patient activation	The intervention did not result in significant differential blood pressure reduction between literacy groups; both groups reduced systolic blood pressure. Greater absolute gains were made in patient activation and hypertension knowledge among the lower literacy group, but the results were not significant.
Heizomi (2020)^44^	Cross-sectional, patients with primary hypertension who were residents of Heris County of Eastern Azerbaijan (Iran)	300	Iranian Health Literacy Questionnaire, functional, communicative, critical	The cognitive and social skills that determine the motivation and ability of individuals to gain access to, understand, and use information in ways that promote and maintain good health.^76^	Sociodemographic, education adherence	Association between communication and decision-making domains of health literacy and medication adherence was significant. For women, high number of children and low family socioeconomic status also accounted for the association.
Ilgaz (2024)^45^	Cross-sectional, patients of 2 family health centers with high CVD risk scores (Turkey)	384	HLS-EU (Turkish), functional, critical	None provided	Sociodemographic, CVD risk (SCORE),^77^ family history of CVD, receipt of risk advice	A higher perception of CVD risk was found among participants with higher health literacy levels than among those with lower health literacy levels.
Lindahl (2020)^46^	Cross-sectional, randomized controlled trial participants with ≥1 CVD risk factor (Sweden)	3459	BHLS, functional	The degree to which individuals understand health information.^78^	Sociodemographic, glucose, waist circumference, carotid artery plaques, CVD risk (Framingham Risk Score^71^ and SCORE^79^)	Body weight, fasting plasma glucose, systolic blood pressure, inner carotid arterial wall thickness, and CVD risk scores were inversely correlated with health literacy.
Medyati (2019)^47^	Cross-sectional, cooks (Indonesia)	80	HLS-EU, functional, critical	None provided	CVD risk (Jakarta Cardiovascular Score^80^), blood pressure, BMI, smoking, diabetes, physical activity	52% were in the “high risk of CVD” category. A trend of increased risk of CVD was found in respondents who had low health literacy levels.
Miller (2023)^48^	Cross-sectional, incarcerated men (United States)	349	NVS, functional	The degree to which individuals have the capacity to obtain, process, and understand basic health information and services needed to make appropriate health decisions.^67,68^	Sociodemographic, CVD risk (Framingham Risk Score^71^), perceived control	Health literacy was a significant predictor of Framingham Risk Score and perceived control.
Muscat (2022)^49^	Nonrandomized experimental study, healthy adults aged 40-50 y recruited online (Australia)	1318	NVS, functional	The skills that enable individuals to obtain, understand, and use information to make decisions and to take actions that will have an impact on health status.^74^	Sociodemographic, hypothetical willingness to increase exercise, willingness to accept medication, perceived severity, perceived life impact, impacts on motivation	Health literacy was not associated with level of worry. Individuals with inadequate levels of health literacy were less willing to increase exercise, more willing to accept medication, and less likely to perceive a 135/85 blood pressure reading as serious than those with adequate levels of health literacy.
Nam and Yoon (2021)^50^	Cross-sectional, patients with physical disabilities who were hypertensive (Korea)	211	Short Korean Health Literacy Scale, functional	The cognitive and social skills that determine the motivation and ability of individuals to gain access to, understand, and use information in ways that promote and maintain good health.^76^	Access to health care, provider–patient interactions, hypertension knowledge, hypertension control self-efficacy, hypertension self-care, sociodemographic	Health literacy was not associated with hypertension self-care but had indirect effects through provider–patient interactions and on hypertension knowledge. Health literacy explained 33% of the variability in provider–patient interactions.
Perez (2015)^51^	Cross-sectional, Hispanic adults with hypertension (United States)	144	NVS, functional	The degree to which an individual has the capacity to obtain, communicate, process, and understand basic health information and services to make appropriate health decisions.^81^	Sociodemographic, acculturation, illness perceptions, hypertension	Moderate relationship between acculturation and health literacy; as acculturation and health literacy increased, perception decreased of how threatening hypertension is as an illness. Participants most commonly attributed hypertension to overweight, stress, inadequate diet, and family history.
Rahimi (2024)^52^	Cross-sectional, health center patients aged 30-65 y (Iran)	248	HELIA, functional, critical	The ability to communicate and understand basic health information to make appropriate health decisions concerning health care and disease prevention.^72^	Sociodemographic, CVD risk (Framingham Risk Score^71^)	Health literacy was significantly inversely associated with Framingham Risk Score.
Ricardo (2014)^53^	Cross-sectional, non-Hispanic White and Black adults aged 21-74 y with mild to moderate chronic kidney disease (United States)	2340	Shortened TOFHLA, functional	The degree to which individuals have the capacity to obtain, process, and understand basic health information and services needed to make appropriate health decisions.^67,68^	Medical history, medications, blood pressure, estimated glomerular filtration rate, LDL, HbA_1c_, self-reported history of CVD	Those with limited health literacy levels were more likely than those with adequate health literacy levels to have lower mean estimated glomerular filtration rate, less likely to achieve blood pressure control, and more likely to self-report a history of CVD. No significant association was found between health literacy levels and HbA_1c_ or LDL cholesterol.
Rose (2014)^54^	Cross-sectional, community-dwelling people with serious mental illness currently receiving care in a community psychiatric treatment program (United States)	98	TOFHLA and short-form REALM, functional	The degree to which individuals have the capacity to obtain, process, and understand basic health information and services needed to make appropriate health decisions.^67,68^	Sociodemographic, health status, health conditions, screening status for diabetes, hypertension and high cholesterol, BMI, blood pressure, physical activity, tobacco use, alcohol use, medication adherence, CVD	The most common sources for receiving health information were health services (46%) and media (45%). As compared with the remainder of the sample, health literacy levels were higher in older people, housewives, individuals with lower education levels, and underweight people. An association was observed between health literacy and site of recruitment.
Soto Mas (2018)^55^	Randomized controlled trial, Spanish-speaking adults (United States)	155	TOFHLA, functional	None provided	Spanish cardiovascular health questionnaire, English-language skills, sociodemographic	Cardiovascular behavior and TOFHLA improvements were significantly greater in the intervention group versus the control group. Health literacy did not influence changes in CVD behavior.
Striberger (2022)^56^	Cross-sectional, patients with intermittent claudication from 3 vascular surgical departments (Sweden)	158	HLS-EU, functional, critical	The cognitive and social skills that determine the motivation and ability of individuals to gain access to, understand, and use information in ways that promote and maintain good health.^76^	Sociodemographic, self-efficacy, quality of life, BMI, blood pressure, cholesterol, glucose, comorbidities, smoking	Patients with sufficient health literacy levels had higher self-efficacy and quality of life than those with insufficient health literacy levels. Half of participants had inadequate or problematic health literacy levels.
Tiller (2015)^57^	Cross-sectional, elderly participants of an East German cohort study (Germany)	1107	Short-form HLS-EU, functional, critical	Knowledge and competence to access, understand, appraise, and apply health information for health judgment.^11^	Sociodemographic, CVD and diabetes status, smoking, alcohol, hypertension, antihypertensive medication, awareness of own hypertension, BMI, quality of life	No association was found between health literacy and blood pressure, body weight, or hypertension. Health literacy was inversely associated with diabetes status.
Tschaftary (2018)^58^	Cross-sectional, patients attending a combined primary care/cardiology practice (Germany)	1039	One scale from HLS-EU, functional, critical	None provided	Sociodemographic, attendance of preventive services, engagement in screening measures, lifestyle changes, willingness to engage in lifestyle change, subjectively estimated CVD risk	Health literacy was inversely associated with likelihood to consider preventive lifestyle measures but not of engaging in them. Patients with reduced health literacy levels asked more frequently for free or subsidized offers even after adjustment for income. Individuals with increased health literacy levels more frequently reported no subjectively perceived need to change lifestyle.
Wang (2017)^59^	Cross-sectional, hypertensive patients in 6 rural towns (China)	882	BHLS, functional	The cognitive and social skills that determine the motivation and ability of individuals to gain access to, understand, and use information in ways that promote and maintain good health.^76^	Sociodemographic, health-related quality of life, self-efficacy for managing chronic disease	Health literacy and self-management efficacy were significantly related to health-related quality of life.
Warren-Findlow (2019)^60^	Pilot intervention, patients with hypertension treated at a low-cost clinic (United States)	52	NVS and 2 medication labels from TOFHLA, functional	The degree to which individuals have the capacity to obtain, process, and understand basic health information and services needed to make appropriate health decisions.^67,68^	Sociodemographic, health status, participant satisfaction, blood pressure, self-reported physical and emotional health, BMI, hypertension self-care behaviors, medication adherence, diet, physical activity, weight management, barriers to medication adherence	Participants reported significant improvements in diet and weight management but not adherence. Those with low NVS scores reported significant improvements in diet, and those with low TOFHLA scores had significant reductions in barriers to medication adherence.
Yardimci Gürel (2024)^61^	Cross-sectional, women (Turkey)	409	HLS-EU (Turkish), functional, critical	The skills that enable individuals to obtain, understand, and use information to make decisions and to take actions that will have an impact on health status.^74^	Sociodemographic, knowledge level of CVD risk factors	A moderate significant correlation was found between health literacy and knowledge of CVD risk.
Zhang (2024)^62^	Cross-sectional, patients with systemic lupus (China)	201	Health Literacy Management Scale, functional, communicative, critical	None provided	Sociodemographic, attitudes and beliefs about CVD risk, social support, anxiety and depression, self-efficacy	A positive correlation was found between health literacy and correct CVD risk perception.
Zhao (2022)^63^	Cross-sectional, patients receiving risk-of-stroke screening (China)	588	Chinese Citizen’s Health Literacy Questionnaire, functional	The ability of individuals to acquire, understand, and deal with necessary health knowledge to improve their health.^81^	Sociodemographic, family function	106 (18.0%) people in the sample reached qualified levels of health literacy.
Zullig (2014)^64^	Pilot experimental, patients with CVD risk factors receiving care from hospital-based primary care clinics (United States)	23	REALM, functional	None provided	Sociodemographic, smoking, financial status, blood pressure, pulse, body weight, medication adherence	Nonsignificant improvements were observed in blood pressure, body weight, and medication adherence. 40% of the sample had low REALM scores.

Abbreviations: BHLS, Brief Health Literacy Screen; BMI, body mass index; CVD, cardiovascular disease; HbA_1c_, hemoglobin A_1c_; HELIA, Health Literacy for Iranian Adults; HLS-EU, European Health Literacy Survey Questionnaire; LDL, low-density lipoprotein; NVS, Newest Vital Sign; REALM, Rapid Estimate of Adult Literacy in Medicine; SCORE, European Systematic Coronary Risk Evaluation; TOFHLA, Test of Functional Health Literacy in Adults.

### Health Literacy Tools

We identified 15 unique instruments as tools for measuring health literacy. Seven of these instruments were performance based and focused on abilities in comprehension, health knowledge, literacy, and numeracy, with prescribed cutoffs for “adequate” and “inadequate” health literacy groups. Seven instruments were self-reports, asking participants to gauge their ease or difficulty in understanding multiple health literacy domains. Finally, 1 tool used a combination of performance and self-reported measures. The Newest Vital Sign (NVS) was the most frequently used measure (n = 6) with the Test of Functional Health Literacy in Adults (TOFHLA) and its short form (n = 6). Except for 1 article, which used an author-constructed tool, all studies used tools validated for their sample populations.

### Studies Describing the Health Literacy of Populations at Risk of CVD

Four studies described the health literacy profiles of populations at risk of CVD. A study by Araújo et al^32^ aimed to describe the health literacy of adults with hypertension and diabetes in Portugal. By applying the multidimensional European Health Literacy Survey, this study found more health literacy barriers among people with comorbidities than among those without comorbidities, as well as sample-wide difficulties in health promotion aspects of health literacy, including understanding information on food packaging, judging media information, and knowing how to prevent or manage “conditions like being overweight, high blood pressure, or high cholesterol.” Also using the European Health Literacy Survey, Striberger et al found that half of the sample’s 158 adults with intermittent claudication (muscle ischemia) had inadequate health literacy levels, and patients with sufficient health literacy levels reported a higher quality of life than those with insufficient health literacy levels.^56^

Similarly, Debussche et al^37^ used the Health Literacy Questionnaire to describe the health literacy of a French population at risk of CVD. Of the 9 health literacy areas measured by the Health Literacy Questionnaire, the following posed the most difficulties: “have sufficient information to manage my health,” “have social support for health,” and “ability to navigate the healthcare system.” In a study concerned with adults at risk of stroke, Zhao et al administered the Chinese Citizen’s Health Literacy Questionnaire and found that 18% of their sample had adequate health literacy levels.^63^

When discussing the implications of their findings, these studies emphasized the usefulness of measuring the health literacy of individuals and populations by (1) using community workers as health literacy promoters, (2) implementing practitioner communication strategies such as teach-back, and (3) assessing the availability and accessibility of health information.^32,37,56,63^

### Studies Implementing Health Literacy Interventions

Eight studies delivered interventions: 3 were patient education programs,^41,55,60^ 3 provided personalized information to patients,^33,39,64^ and 2 were delivered at a clinic level.^40,43^

Of the interventions that provided education programs directly to participants, we observed some changes in outcome measures. Participants enrolled in an English course had improved TOFHLA scores and cardiovascular behaviors once health literacy was embedded into their curriculum, independent of English proficiency.^55^ In a curriculum-based intervention by Warren-Findlow et al, significant diet improvements occurred among those who initially scored low on the NVS, and fewer barriers to medication adherence were reported among those with lower TOFHLA scores than among those with higher TOFHLA scores.^60^ Participants with lower health literacy levels in the educational sessions of Fu et al^41^ on proper home monitoring of blood pressure had greater weight loss than their control counterparts.

A pharmacist-led intervention to improve medication adherence revealed that 40% of the sample had low scores on the Rapid Estimate of Adult Literacy in Medicine, and use of individualized calendars with instructions increased medication adherence in a sample of patients with CVD risk factors; however, the Rapid Estimate of Adult Literacy in Medicine was not measured postintervention.^64^ In a web-based decision aid trial, Bonner et al^33^ noted that those who received a literacy-sensitive aid were more likely than those who did not receive one to know their risk of CVD at follow-up. Yet, risk perceptions and intentions did not differ significantly between groups. A similar text message intervention by Fajardo et al^39^ found differing results by health literacy level, which were partially alleviated with more specific nudge-style messaging.

In a clinic-level intervention, Faruqi et al^40^ reported increases in recording of patient CVD risk factors and health literacy practices at an organizational level; however, the differences were not significant. At clinics participating in a quality improvement intervention, patients who were hypertensive had a mean reduction in systolic blood pressure, although these differences were not significant by health literacy level.^43^

Although the intervention studies measured various outcomes, these studies all reported improvements, with only 1 study observing no significant differences between health literacy groups. When discussing implications of their studies, these articles mentioned the validity of their model, the need for further research into effective interventions, and a need to increase CVD awareness in groups susceptible to CVD.^33,39,40,41,43,55,60,64^

### Studies Investigating Health Literacy’s Association With CVD Risk

Most studies investigated a role for health literacy in CVD risk (Table 2). Of 23 studies, 20 found some relationship between health literacy level and their chosen CVD measure. Most studies (n = 11) investigated the role of health literacy in determining objective CVD risk, while 9 studies assessed the role of health literacy in attitude, knowledge, and beliefs toward CVD. Three studies assessed objective risk and CVD perceptions.

Of 14 studies that assessed a role for health literacy in objective CVD risk, 7 revealed significant associations; 4 found partial associations, where health literacy predicted some but not all outcomes measured; and 3 demonstrated no associations. The most common risk factors measured were blood pressure (n = 14 studies), smoking/tobacco use (n = 13 studies), body mass index (n = 12 studies), and self-reported physical activity (n = 12 studies). Seven studies measured CVD event risk with composite tools, including 4 that used the Framingham Risk Score or a culturally modified version of it. Regardless of the strength or significance of these findings, all studies that showed an association between health literacy levels and objective CVD risk were positive when measuring a favorable outcome (ie, blood pressure control) and inverse when measuring an unfavorable outcome (ie, CVD mortality, presence of CVD risk factors).

Of 12 studies that investigated the role of health literacy in attitudes, knowledge, and beliefs about CVD, 11 found some relationship between the factors, although the extent of the relationship varied, with some articles showing a less pronounced link than others. The most common domains assessed were attitudes toward CVD risk (n = 7 studies), knowledge (n = 5 studies), and intention of behavior change (n = 2 studies). While CVD knowledge showed a clear association with health literacy in these studies, relationships between health literacy and attitudes toward CVD risk had a less consistent direction. Approximately half of these studies adapted their attitude measures from existing questionnaires, with the other half either creating their own measure or not specifying a source of adaptation. Where participant knowledge levels were measured, questions were usually adapted from consumer information released by the health authority in the study’s area.

### Risk of Bias

The mean (SD) OHAT risk of bias in studies was 7.26 (1.8). The lowest risk score was 3 (n = 2 studies) and the highest risk score was 11 (n = 2 studies) (Table 3), suggesting that most studies had at least 1 area at risk of bias. Specific criteria with the highest risk of bias were selection of participants resulting in appropriate comparison groups (mean, 1.3 [SD, 0.7]) and completeness of data without attrition or exclusion from analysis (1.2 [0.6]). These results indicate that comparison groups in studies, often low versus high health literacy, may have had baseline characteristics that differed beyond the exposure of interest (ie, health literacy). While this risk of bias is difficult to minimize because of the contextual nature of health literacy, Tiller et al^57^ mitigated much of this risk of bias by using a multistep recruitment strategy, and Zullig et al^64^ mitigated this risk by randomly selecting participants from electronic health records. Although few studies were at higher risk of bias than other studies in accounting for important confounders and modifiers, several studies were at high risk of bias, indicating that insufficient evidence was shown to demonstrate the inclusion or justified exclusion of potential confounders in statistical analysis. Similarly, several studies failed to clearly report why participants were excluded from analysis, meaning that the completeness of outcome data cannot be fully ascertained.

**Table 3.** Results of Office of Health Assessment and Translation Risk of Bias Tool analysis of studies in a scoping review on cardiovascular disease risk and health literacy

	Question^a^												
Author (year)	1	2	3	4	5	6	7	8	9	10	11	12	Total^b^
Adam (2023)^30^	1	0	2	1	1	1	0	NA	NA	NA	NA	NA	6
Aranha (2015)^31^	2	1	1	1	1	1	1	NA	NA	NA	NA	NA	8
Araújo (2018)^32^	1	0	0	1	1	0	0	NA	1	0	1	1	6
Bonner (2022)^33^	2	2	1	1	0	1	1	NA	NA	NA	NA	NA	8
Cheng (2018)^34^	1	0	2	1	1	0	0	NA	NA	NA	NA	NA	5
Crook (2016)^35^	1	1	2	1	1	1	1	NA	NA	NA	NA	NA	8
Darvishpour (2022)^36^	0	1	2	1	1	1	0	NA	0	0	1	1	8
Debussche (2018)^37^	1	2	1	1	1	1	1	NA	NA	NA	NA	NA	8
Enjezab (2021)^38^	1	0	1	1	1	0	0	NA	0	0	1	1	6
Fajardo (2024)^39^	1	1	3	1	1	1	1	1	NA	NA	NA	NA	9
Faruqi (2015)^40^	2	2	0	1	1	1	1	NA	NA	NA	NA	NA	8
Fu (2022)^41^	2	1	1	1	0	1	1	NA	NA	NA	NA	NA	7
Gaffari-Fam (2020)^42^	1	1	1	1	1	2	1	1	NA	NA	NA	NA	8
Halladay (2017)^43^	2	1	2	1	1	2	2	NA	NA	NA	NA	NA	11
Heizomi (2020)^44^	2	1	1	0	1	0	1	NA	NA	NA	NA	NA	6
Ilgaz (2024)^45^	1	1	1	1	1	1	1	NA	NA	NA	NA	NA	7
Lindahl (2020)^46^	2	2	1	1	1	1	1	NA	NA	NA	NA	NA	9
Medyati (2019)^47^	0	1	2	0	1	1	1	NA	NA	NA	NA	NA	6
Miller (2023)^48^	1	1	1	1	1	1	1	NA	NA	NA	NA	NA	7
Muscat (2022)^49^	2	1	1	1	1	1	1	NA	NA	NA	NA	NA	8
Nam (2021)^50^	1	0	0	1	1	0	0	NA	NA	NA	NA	NA	3
Perez (2015)^51^	0	0	1	1	1	1	1	NA	NA	NA	NA	NA	5
Rahimi (2024)^52^	1	1	1	1	1	1	1	NA	NA	NA	NA	NA	7
Ricardo (2014)^53^	1	2	1	1	1	1	1	NA	NA	NA	NA	NA	8
Rose (2014)^54^	2	1	1	1	1	1	1	NA	NA	NA	NA	NA	8
Soto Mas (2018)^55^	2	1	1	1	1	1	1	NA	NA	NA	NA	NA	8
Striberger (2022)^56^	0	2	2	1	2	1	1	NA	NA	NA	NA	NA	9
Tiller (2015)^57^	1	0	2	1	1	1	0	NA	NA	NA	NA	NA	6
Tschaftary (2018)^58^	2	1	1	1	1	1	1	NA	NA	NA	NA	NA	8
Wang (2017)^59^	3	1	1	1	2	1	2	NA	NA	NA	NA	NA	11
Warren-Findlow (2019)^60^	1	0	0	1	1	0	0	NA	1	0	1	1	6
Yardimci Gürel (2024)^61^	2	2	1	1	0	1	1	NA	NA	NA	NA	NA	8
Zhang (2024)^62^	1	0	2	1	1	0	0	NA	NA	NA	NA	NA	5
Zhao (2022)^63^	1	1	1	1	1	1	0	NA	NA	NA	NA	NA	6
Zullig (2014)^64^	1	1	2	1	1	1	1	NA	NA	NA	NA	NA	8

Abbreviation: NA, not applicable.

a Numbers in column headers correspond to the following questions asked: (1) Was administered dose or exposure level adequately randomized? (2) Was allocation to study groups adequately concealed? (3) Did selection of study participants result in appropriate comparison groups? (4) Did the study design or analysis account for important confounding and modifying variables? (5) Were experimental conditions identical across study groups? (6) Were the research personnel and human subjects blinded to the study group during the study? (7) Were outcome data complete without attrition or exclusion from analysis? (8) Can we be confident in the exposure characterization? (9) Can we be confident in the outcome assessment? (10) Were all measured outcomes reported? (11) Were there no other potential threats to internal validity (eg, statistical methods were appropriate and researchers adhered to the study protocol)? (12) Did the study design or analysis account for important confounding and modifying variables (including unintended coexposures) in experimental studies?

b Risk of Bias Tool scores: 0 = definitely low risk of bias, 1 = probably low risk of bias, 2 = probably high risk of bias, 3 = definitely high risk of bias.

## Discussion

Our review identified 3 major themes of studies: studies that described populations at risk of CVD, interventions with population groups at risk for CVD, and investigations of the association between health literacy and CVD risk. Descriptive studies found multiple health literacy barriers and facilitators in their samples, and interventions showed feasibility in improving CVD risk factors and knowledge. However, description and intervention studies were less numerous than descriptive studies, likely because these studies often included broad cross sections of the population without a particular disease focus or were conducted at a grassroots level by following processes such as Optimising Health Literacy and Access, where local health literacy needs are assessed, responded to, and monitored over time.^12^ Future reviews concerned with descriptive health literacy studies may benefit from including nonscholarly literature in their search strategies.

The consistent association between health literacy and objective CVD risk that we observed in the studies suggests that health literacy plays a role in determining one’s CVD risk. Given the extensive literature linking socioeconomic disparities and CVD risk factors,^9,10^ results from our study suggest further exploration of the role of health literacy as a possible mediator of these links. Although previous associations were drawn between health literacy and elements of CVD risk (ie, age, sex),^1-5^ our review found a similarly consistent relationship when composite risk scores were used. This relationship was also observed in studies investigating health literacy’s association with attitudes, knowledge, and beliefs. However, these studies were heterogenous in design, suggesting a less stable evidence base than objective CVD risk. Studies investigating the association of the NVS with CVD risk may be sufficient to warrant a future meta-analysis. While a meta-analysis would provide additional rigor, the functional-only scope of the NVS is limited. Future studies may be able to expand the applicability of these findings by measuring the full spectrum of health literacy with a multidimensional tool.

Ambiguity in the definition of health literacy is an issue, with no field consensus established and 10 of the 35 studies providing no definition for the term. Although authors’ conceptualization may be partially inferred through their choice of measurement tool, their position in this vast field has not been fully communicated. Additionally, studies overwhelmingly recruited patient participants, which may have overlooked the health literacy needs of those not engaged in a health service, potentially introducing care-seeking bias. Future studies could use population-based sampling, taking note to measure the communicative and critical elements of health literacy, which rely on adequate access and supports.

### Strengths and Limitations

Our review had several limitations. First were the limitations of the studies identified through risk-of-bias analysis (eg, selection of participants, completeness of data). Second, the cross-sectional design of most studies limited our ability to draw associations between health literacy and CVD. Third, studies were homogenous in location, recruitment setting, and number of participants. Fourth, although each study was not reviewed by 2 authors, a second reviewer randomly screened 10% of articles at each stage, including the pilot stage. This process allowed for a second reviewer when interrater agreement fell below 80%, which it never did and the second reviewer was not needed.

Fifth, the scope of this review was a limitation; as such, we may not have captured information on preventive practices or attitudes (eg, knowledge and perceptions of physical activity) that apply to several noncommunicable diseases or to sociodemographic risk factors such as age and sex. However, to our knowledge, this study is the first to describe the scope of the relationship between health literacy and CVD prevention and to use a robust tool to assess studies’ risk of bias. Future studies may expand these findings by conducting a meta-analysis on homogenous sections in these findings (eg, relationship between NVS and the Framingham Risk Score).

## Conclusions

This review found that most health literacy studies concerned with CVD prevention were cross-sectional and used risk predictor tools such as the Framingham Risk Score or stand-alone measurements such as blood pressure. Study designs were homogenous, highlighting a need for novel health literacy studies that use longitudinal methods. Findings from this study indicate that clinicians assessing and responding to a patient’s risk of CVD would benefit from considering patient health literacy in this process. Similarly, policy makers are likely to benefit from applying a health literacy lens to CVD prevention strategies.
